# Prevalence of Older Hospitalised Adults with Sustained Fractures after a Fall in Regional Australian Hospitals

**DOI:** 10.3390/healthcare12131318

**Published:** 2024-07-01

**Authors:** Emma Holden, Ruth Devin, Joyita Bhattacharya, Frances Waldie, Isabel Watt, Chiung-Jung (Jo) Wu

**Affiliations:** 1 Medical Services Group, Sunshine Coast Hospital and Health Service, Birtinya, QLD 4575, Australia; emma.holden2@health.qld.gov.au (E.H.); ruth.devin@health.qld.gov.au (R.D.); joyita.bhattacharya@health.qld.gov.au (J.B.); frances.waldie@gmail.com (F.W.); 2 Central Adelaide Local Health Network, University of Adelaide, Adelaide, SA 5000, Australia; isabel.watt@uqconnect.edu.au; 3 School of Health, University of Sunshine Coast, Petrie, QLD 4502, Australia; 4 Royal Brisbane and Women’s Hospital, Herston, QLD 4006, Australia

**Keywords:** older people, fall, regional hospital

## Abstract

Falls commonly occur in hospitals, particularly among older adults. Fractures in the older population can cause major morbidity, which can result in long hospital admissions and increased care costs. This study aimed to characterise the demographics of patients aged 65 years and over who fell in hospital and to determine the type of fractures they sustained. A descriptive study was undertaken to examine hospital data of older inpatients who had a fall during admission in two regional Queensland hospitals in Australia over a 2.5-year period. The prevalence of inpatient falls was 1.28%. Most falls were unwitnessed (77.34%) and they had an average of seven medical comorbidities. The mean age was 80.4 years and 63% were male. Women who fell were significantly older than men (*p* = 0.004). The mean length of stay of in-hospital fallers was 22.77 days and same admission mortality was 9.3%. Thirty-three fall events (3.8%) resulted in fractures, some with multiple injuries. The most common fracture was neck of femur, followed by rib, femur, and facial fractures. In conclusion, this study identifies the incidence of falls increased with age, most falls were unwitnessed, as well as provides evidence that patients with falls had multiple comorbidities and long hospital admissions. The data could be used to optimise fall prevention strategies and to refine post-fall assessment pathways.

## 1. Introduction

A fall is an event whereby a person comes to rest on the ground or a lower level inadvertently [1]. Falls are the most common cause of hospitalisation due to injury in Australia, with 233,000 hospitalizations in 2021–2022 [2]. Adults over the age of 65 years are more likely to sustain an injury requiring hospitalisation than younger groups and are at higher risk of death due to complications following a fall [2]. Older women are more likely to sustain a fracture than older men [3] and the most common type of fracture sustained by women who fall in the community are wrist fractures, followed by humerus, vertebral, and hip fractures [4]. The most common injuries requiring hospitalisation following a fall in the community are fractures, followed by open wounds and soft tissue injuries [2], and the most common site of fracture requiring hospital admission was the hip and femur [3].

Most falls occur at home or in residential aged care facilities [3,4], however, in Australia, there were 48.5 hospitalizations per 100,000 population due to falls that occurred in healthcare settings in the 2019–2020 period [3]. Known risk factors for falls within hospitals include advanced age, male sex, previous falls, urinary incontinence, confusion, orthostatic hypotension, and polypharmacy especially if it includes sedative medications [5,6]. Previous studies have shown the most common bony injury following an inpatient fall is hip fracture [7,8].

The cost to the health system of managing injuries is significant, with $10.3 billion (7.7%) of Australian healthcare spending going towards management of injuries in 2019–20, and of this, $4.3 billion managing the consequences of falls [3]. The average length of stay in Australian hospitals following a fall-related injury is 7.1 days, compared to 4.7 days for all injuries [2]. While the monetary costs of managing the complications of falls are great, the cost to the patient and their families due to prolonged admission, pain, fear, loss of confidence, and deterioration in mobility and function can be far greater.

A variety of fall risk reduction strategies should be undertaken within health facilities to optimise fall prevention for inpatients, including within organizational, staff, patient, and environment categories [9]. Multifactorial fall prevention strategies with components targeting exercise, medication optimisation, management of urinary incontinence, environment optimisation, and information provision, have been shown to reduce the rate of falls of patients in hospitals [10,11].

There are multiple fall prevention strategies available, including the Queensland “Stay On Your Feet” initiative [12]. There is also an inpatient fall assessment and management plan [13] that is widely used within Queensland hospitals in Australia, with the expectation that it is to be completed within eight hours of patient admission. This tool aims for early identification of risk factors for falls, including previous falls, unsteadiness, incontinence, polypharmacy, confusion, or delirium. It also prompts for actions from the nursing staff, including referral for physiotherapy review, toileting routine, and notification of the medical officer of high fall risk with the aim of reducing the risk of in-hospital falls. However, there are limited data delineating the characteristics of patients who fall within regional Queensland hospitals and the bony injuries they sustain.

### Aims

This study aimed to establish the characteristics of older patients (aged 65 years and older) who fell while admitted to two regional hospitals in a medical or surgical setting over a 2.5-year period. This study also aimed to determine the incidence of fractures and the types of fractures that were sustained during these fall events.

## 2. Materials and Methods

### 2.1. Study Design, Participants, and Setting

A retrospective review of data from two regional hospitals (Sunshine Coast University Hospital and Nambour General Hospital) in Queensland, Australia, was conducted. Inclusion criteria included patients who were aged 65 years and older, who had a fall while admitted under a medical or surgical team at the two participating hospitals during the study period from February 2018 to July 2020. Exclusion criteria included episodes not meeting the World Health Organization definition of a fall [1], episodes that occurred when the patient was located in an emergency, short stay, transit lounge, or day therapy unit, and episodes that occurred while the patient was admitted to the “Hospital in the Home” program.

### 2.2. Data Collection

Data were collected after ethics approval was obtained. Inpatient fall events were identified using the Queensland Government information reporting tools [14]. Data were collected from electronic medical records by five trained data collectors using a medical audit tool [15] and included demographic information, comorbidities, type of injury sustained, length of stay, and mortality outcome.

### 2.3. Statistical Analyses

Data were analysed using IBM Statistical Package for the Social Science version 27. Independent *t*-test was used to determine relationships to age. Chi-squire test was used to examine the differences of sex, according to fracture or non-recorded fracture. Statistical significance was determined by *p* < 0.05.

### 2.4. Ethics Considerations

This clinician-initiated study was approved by the Prince Charles Hospital Human Research Ethics Committee (LNR/2020/QPCH/65743). Data were accessed by research personnel only. Data were kept on a hospital-secured server in a private medical office.

## 3. Results

### 3.1. Characteristics

The total number of medical and surgical admissions in the 65 years and older population to both hospitals during this period was 55,414, with an incidence of at least one fall during a hospital admission of 1.28% in this older age group. There was a total of 874 fall events that met the criteria for inclusion, with 167 events (19.1%) being repeat fall events during a hospital admission.

The mean age for males was 79.78 years and 81.36 years for females. Women who fell were significantly older than men (*p* = 0.004). The incidence of falls increased with age, from 0.68% in the 65–69 years age group to 2.22% in the 95+ years age group. The co-morbidities of all patients who fell ranged from 0–16, and for those who sustained fractures was 3–15. Delirium was documented in 39.5% of patients who fell and was likely present (on review of clinical notes) but not documented in a further 17.4% of patients. Most patients who fell had an inpatient falls assessment and management plan completed prior to the fall (96.8%). See Table 1.

### 3.2. Falls

Males over the age of 65 years are 1.7 times more likely to fall in hospital than females of the same age. This is most pronounced in younger fallers (males 65–69 years are 2.7 times more likely than females to fall). However, it is not observed in the over-90-year population, with males in the 90–94 year and 95+ year age groups being 1.1 times more likely to fall than females.

Falls were unwitnessed in 676 events (77.34%). Most occurred from standing height (n = 459), with the remainder being from unknown height (n = 237), out of bed (n = 145) and sitting height (n = 125). There was no correlation to the time of day that falls occurred. Thirty-nine fall events (4.5%) resulted in fractures, with four patients sustaining multiple fractures. See Table 2. The total number of fractures was 45. The most common fracture was neck of femur (n = 11), followed by ribs (n = 7), spine (n = 5), femur (n = 4), and facial (n = 4). Nineteen patients proceeded to surgical management of their fractures. Two patients required blood transfusions and 13 patients required other interventions, including semirigid C-spine collars, casts, slings, withholding anticoagulation, moon boots, sinus precautions, and antibiotics.

Eighty-four patients died during their hospital admission which included a fall (incidence 9.6%).

The mean length of stay for fallers was 22.77 days, compared to the mean length of stay for all patients 65 years or older admitted to inpatient medical and surgical units during that period of 3.88 days. There was a re-admission rate within 30 days of discharge of 19.2% for patients who fell during their hospital admission.

There were no significant differences between fractures or non-recorded fractures according to sex, witnessed fall, and fall height. See Table 3.

## 4. Discussion

Falls in older inpatients are common, with an incidence of 1.28% in all patients over 65 years. The incidence increases with age, with more than 2% of patients over the age of 95 years falling during a hospital admission. Males are more likely to fall than females, but this trend does not occur with patients beyond the age of 90 years when both males and females are equally likely to fall. This is consistent with previous studies demonstrating older age and male sex as risk factors for falls [5,6].

Over half of the patients who fell in hospital had either delirium documented or likely delirium present on review of their clinical notes. This identifies the possible role of delirium prevention and management as a technique for reducing the risk of inpatient falls.

This has also been supported by previous studies [16,17]. A recent original study revealed the significant relationships between delirium and in-hospital falls [15]. Similarly, a meta-analysis showed approximately a 40% reduction of fall risks in the delirium prevention program [16]. However, the challenges of developing a delirium prevention intervention to reduce in-hospital falls in clinical practice are raised [16]. Future clinical research should consider not only older hospitalised people’s demographic characteristics but also the feasibility of multifactorial and multiple components of the intervention.

The time of day did not impact the likelihood of a fall occurring. This may reflect that although there is less nursing staff overnight, fewer patients are attempting to mobilise at these times. This contrasts with a previous study showing that the time of falls peaks between 04:00 a.m. and 07:00 a.m. [18]. Further research into the location of falls and activities being undertaken at the time of fall could be used to hone fall prevention strategies further.

The most common fracture sustained after an inpatient fall was neck of femur. This is in contrast with the data from community populations demonstrating the most common fracture type following falls was wrist fractures [4]. Hip fractures are associated with high morbidity and mortality. 98% of Australian patients presenting with hip fractures require surgical management, only 44% return to their baseline level of mobility at 120 days [19], and the mortality rate at 30 days is 8.2% [20].

The incidence of death during admission was 9.6% for all older inpatient fallers. This compares to the mortality rate for all patients admitted to Australian hospitals for fall-related injuries of 122 per 100,000 admissions (0.12%) [3]. Within Queensland hospitals, the in-hospital mortality rate of patients over the age of 65 years admitted with serious injury was 5% [21]. Given that the patients already required hospital admission for medical or surgical reasons prior to the falls, the higher mortality rate within this study may be due to other medical and surgical issues rather than directly related to the fall.

Patients who fell in hospital had a far longer length of stay than that of all patients of the same age group admitted to surgical and medical units within these hospitals. This is consistent with previous studies, which demonstrate that falls during admission, both resulting in injury or not, result in prolonged length of stay [22,23]. This may be due to factors such as the severity of acute illness impacting the risk of falling, presence of delirium, and mobility and functional decline following the fall. The cost to the health system associated with a long length of stay following in-hospital falls is significant and highlights the importance of continued work to optimise fall prevention strategies in hospitals.

### Strengths and Limitations

Strengths of the study include that the patients recruited were admitted across a broad range of surgical and medical inpatient units at two regional Queensland, Australia hospitals. It is unclear as to whether there are events that are not reported using these systems, which could result in the total number of falls being underestimated. However, it is the standard culture and expectation at these two hospitals for falls to be routinely recorded as a clinical incident report.

Limitations of this clinical project include its retrospective design, and that the data collected was documented by others. This clinician-initiated study recruited data from two regional hospitals and therefore we acknowledge a small sample. This can impact the accuracy of the results, as shown by the number of patients who had a fall that, on review of their clinical notes, likely had delirium during their hospital admission but did not have the delirium diagnosis formally documented during their admission. Data on some established risk factors for falls, such as polypharmacy and sedative medication use, were not gathered during this study.

Data were not collected on the discharge destination after the admission involving a fall. Risk factors for discharge to a residential aged care facility following admission with injury include older age, greater injury severity, longer hospital stay, and injuries resulting from falls rather than other mechanisms [19]. Further research could establish if these risk factors in community falls are consistent with patients sustaining injury from in-hospital falls.

This study adds information to allow for early identification of those older hospitalised patients who are at risk of falls during their admission within regional hospitals and could be used to target fall prevention interventions to patients who will benefit most. Identification of the most common fracture sites from inpatient falls also suggests potential for the education of junior doctors in targeted examinations of patients’ post-fall, to identify the most common injuries, and to refine post-fall assessment pathways.

## 5. Conclusions

Falls are a common complication of hospital admission for older adults. This study identifies that for patients at two regional Queensland hospitals, the incidence of falls increased with advanced age and that males were more likely to fall than females. Most falls were unwitnessed; however, the time of the day did not affect the likelihood of a fall occurring. The length of stay for patients who fell during their admission was significantly longer than all admissions during the same time period. A fall during admission also portends a poor prognosis, with 9.6% mortality during the admission. The information gathered during this study can be used to target fall prevention interventions for those who are most likely to fall in our hospitals and to refine post-fall assessments.

## Figures and Tables

**Table 1:** Sample characteristics (n = 874 falls).

Variable	Number	%
Sex and age		64.9
Males	567	
65–74	144	
75–84	254	
85+	169	
Females	307	35.1
65–74	74	
75–84	112	
85+	121	
Second or subsequent fall during single admission		
Yes	167	19.1
No	707	80.9
Fall witnessed		
Yes	198	22.7
No	676	77.3
Fall height		
Standing	420	48.1
Sitting	125	14.3
Out of bed	143	16.4
Unknown	186	21.2
Falls resulting in fracture		
No	835	95.5
Yes	39	4.5
Delirium during admission, prior to the fall		
Yes (documented)	377	43.1
No (absent)	345	39.5
Probable—notes suggested but ‘delirium’ not mentioned	152	17.4
Fall risk assessment completed		
Yes	846	96.8
No	28	3.2
Readmission within 30 days		
Yes	167	19.1
No	707	80.9
Death during admission		
Yes	84	9.6
No	790	90.4

**Table 2:** Site of fracture (n = 45).

Site	Number	%
Neck of femur	11	24.4
Ribs	7	15.6
Spine	5	11.1
Femur	4	8.9
Facial	4	8.9
Forearm	3	6.7
Pelvis	2	4.4
Clavicle	2	4.4
Skull	2	4.4
Hand	2	4.4
Foot	1	2.2
Lower leg	1	2.2
Sternum	1	2.2

**Table 3:** Comparison of sex, witnessed, and fall height between factures or non-recorded fractures (n = 874).

Variable	Fracture		*p*
	Yes	No	
	Number (%)	Number (%)	
Sex			*p = 0.335*
Males	24 (4.2)	543 (95.8)	
Females	9 (2.9)	298 (97.1)	
Fall witnessed			*p = 0.257*
Witnessed	5 (2.5)	194 (97.5)	
Unwitnessed	28 (4.3)	647 (95.7)	
Fall height			*p = 0.110*
Standing	13 (3.1)	459 (96.9)	
Sitting/out of bed	8 (6.2)	145 (93.8)	
Unknown	12 (6.5)	237 (93.5)	

## Data Availability

Data are contained within the article.

## References

[1] World Health Organization Falls 2022. https://www.who.int/news-room/fact-sheets/detail/falls.

[2] Australian Institute of Health and Welfare (2023). Falls. Canberra: AIHW. https://www.aihw.gov.au/reports/injury/falls.

[3] Australian Institute of Health and Welfare (2022). Falls in Older Australians 2019–20: Hospitalisations and Deaths among People Aged 65 and Over. Canberra: AIHW. https://www.aihw.gov.au/reports/injury/falls-in-older-australians-2019-20-hospitalisation/contents/summary.

[4] Sanders K.M., Lim K., Stuart A.L., Macleod A., Scott D., Nicholson G.C., Busija L. (2017). Diversity in fall characteristics hampers effective prevention: The precipitants, the environment, the fall and the injury. Osteoporos. Int..

[5] Morris R., O’Riordan S. (2017). Prevention of falls in hospital. Clin. Med..

[6] Montero-Odasso M.M., Kamkar N., Pieruccini-Faria F., Osman A., Sarquis-Adamson Y., Close J., Hogan D.B., Hunter S.W., Kenny R.A., Lipsitz L.A. (2021). Evaluation of clinical Practice Guidelines on Fall Prevention and Management for Older Adults: A Systematic Review. JAMA Netw. Open..

[7] AlSumadi M., AlAdwan M., AlSumadi A., Sangani C., Toh E. (2023). Inpatient Falls and Orthopaedic Injuries in Elderly Patients: A Retrospective Cohort Analysis From a Falls Register. Cureus.

[8] Vaishya R., Vaish A. (2020). Falls in Older Adults are Serious. Indian J. Orthop..

[9] Taylor E., Hignett S. (2016). The SCOPE of Hospital Falls: A Systematic Mixed Studies Review. HERD Health Environ. Res. Des. J..

[10] Morris M.E., Webster K., Jones C., Hill A.M., Haines T., McPhail S., Kiegaldie D., Slade S., Jazayeri D., Heng H. (2022). Interventions to reduce falls in hospitals: A systematic review and meta-analysis. Age Ageing.

[11] Cameron I.D., Dyer S.M., Panagoda C.E., Murray G.R., Hill K.D., Cumming R.G., Kerse N. (2018). Interventions for preventing falls in older people in care facilities and hospitals. Cochrane Database Syst. Rev..

[12] Queensland Health (2022). Stay. On Your Feet: Queensland Health. https://www.health.qld.gov.au/stayonyourfeet/for-hosp-facility.

[13] Queensland Health (2021). In-Patient Falls Assessment and Management Plan. In: Queensland Health, Editor. https://www.health.qld.gov.au/__data/assets/pdf_file/0035/432998/falls-assessment.pdf.

[14] Queensland Government Safety an Quality Education Program: Risk Management. Last Updated 2023. https://csds.qld.edu.au/safetyandquality/risk-management/.

[15] Wu C.J., Chang A.M. (2008). Audit of patients with type 2 diabetes following a critical cardiac event. Int. Nurs. Rev..

[16] He S., Rolls K., Stott K., Shekhar R., Vueti V., Flowers K., Moseley M., Shepherd B., Mayahi-Neysi M., Chasle B. (2022). Does delirium prevention reduce risk of in-patient falls among older adults? A systematic review and trial sequential meta-analysis. Australas. J. Ageing.

[17] Kalivas B., Zhang J., Harper K., Thomas M.K., Dulin J., Marsden J., Robbins P., Hunt K.J., Mauldin P.D., Moran W.P. (2023). The Association between Delirium and In-Hospital Falls: A Cross-Sectional Analysis of a Delirium Screening Program. J. Aging Res..

[18] Kobayashi K., Imagama S., Inagaki Y., Suzuki Y., Ando K., Nishida Y., Nagao Y., Ishiguro N. (2017). Incidence and characteristics of accidental falls in hospitalizations. Nagoya J. Med. Sci..

[19] Australian and New Zealand Hip Fracture Registry (2023). Annual Report. https://anzhfr.org/registry-reports/.

[20] Australian and New Zealand Hip Fracture Registry (2022). Annual Report. https://anzhfr.org/wp-content/uploads/sites/1164/2022/08/ANZHFR-2022-Annual-Report-Full-e-Report-v2.pdf.

[21] Rai S., Brace C., Ross P., Darvall J., Haines K., Mitchell I., van Haren F., Pilcher D. (2023). Characteristics and outcomes of very elderly patients admitted to intensive care: A retrospective multicentre cohort analysis. Crit. Care Med..

[22] Najafpour Z., Godarzi Z., Arab M., Yaseri M. (2019). Risk factors for falls in hospital in-patients: A prospective nested case sontrol Study. Int. J. Health Policy Manag..

[23] Kalivas B., Zhang J., Harper K., Dulin J., Heincelman M., Marsden J., Hunt K.J., Mauldin P.D., Moran W.P., Thomas M.K. (2023). The combined effect of delirium and falls on length of stay and discharge. J. Healthc. Qual..

